# American Dental Association

**Published:** 1887-04

**Authors:** 


### AMERICAN DENTAL ASSOCIATION.

The letter of Dr. Field, in this number, will serve to call attention to a matter which will, of course, receive the attention of the Executive Committee of the American Dental Association. We do not think it advisable to forego the meeting of the Association for anything whatever, but some will desire a change of time and place.

